# Clinical outcomes of recurrent or metastatic head and neck cancer after failure of platinum and nivolumab: a multicenter retrospective study

**DOI:** 10.1093/oncolo/oyaf018

**Published:** 2025-03-31

**Authors:** Takane Watanabe, Hiroki Oka, Kengo Nagashima, Hideaki Nishi, Yoshihiko Kumai, Hiroaki Iijima, Kenji Okami, Yasushi Shimizu, Satoshi Kano, Kazue Ito, Tomoko Yamazaki, Hideaki Takahashi, Nobuhiko Oridate, Tomoya Yokota, Taiji Koyama, Naomi Kiyota, Yasuyoshi Sato, Shunji Takahashi, Kyoko Kato, Shigenori Kadowaki, Yoshitaka Honma

**Affiliations:** Department of Head and Neck, Esophageal Medical Oncology, National Cancer Center Hospital, 104-0045, 5-1-1, Tsukiji, Chuo-ku, Tokyo, Japan; Department of Head and Neck, Esophageal Medical Oncology, National Cancer Center Hospital, 104-0045, 5-1-1, Tsukiji, Chuo-ku, Tokyo, Japan; Biostatistics Unit, Clinical and Translational Research Center, Keio University Hospital, Tokyo 160-0016, Japan; Otorhinolaryngology/Head and Neck Surgery, Nagasaki University Hospital, Nagasaki 852-8501, Japan; Otorhinolaryngology/Head and Neck Surgery, Nagasaki University Hospital, Nagasaki 852-8501, Japan; Otorhinolaryngology/Head and Neck Surgery, Tokai University Hospital, Isehara 259-1193, Japan; Otorhinolaryngology/Head and Neck Surgery, Tokai University Hospital, Isehara 259-1193, Japan; Department of Medical Oncology, Hokkaido University Hospital, Sapporo 060-8648, Japan; Otorhinolaryngology, Head and Neck Surgery, Hokkaido University Hospital, Sapporo 060-8648, Japan; Head and Neck Oncology, Miyagi Cancer Center, Natori 981-1239, Japan; Head and Neck Oncology, Miyagi Cancer Center, Natori 981-1239, Japan; Otorhinolaryngology, Yokohama City University Hospital, Yokohama 236-0004, Japan; Otorhinolaryngology, Yokohama City University Hospital, Yokohama 236-0004, Japan; Gastrointestinal Oncology Department, Shizuoka Cancer Center, Shizuoka 411-8777, Japan; Department of Medical Oncology and Hematology, Kobe University Hospital, Kobe 650-0017, Japan; Department of Medical Oncology and Hematology, Kobe University Hospital, Kobe 650-0017, Japan; Department of Medical Oncology, The Cancer Institute Hospital of Japanese Foundation for Cancer Research, Tokyo 135-8550, Japan; Department of Medical Oncology, The Cancer Institute Hospital of Japanese Foundation for Cancer Research, Tokyo 135-8550, Japan; Department of Clinical Oncology, Aichi Cancer Center Hospital, Nagoya 464-8681, Japan; Department of Clinical Oncology, Aichi Cancer Center Hospital, Nagoya 464-8681, Japan; Department of Head and Neck, Esophageal Medical Oncology, National Cancer Center Hospital, 104-0045, 5-1-1, Tsukiji, Chuo-ku, Tokyo, Japan

**Keywords:** salvage-line chemotherapy, recurrent or metastatic head and neck cancer, immune checkpoint inhibitor, nivolumab, long-term survival, best supportive care

## Abstract

**Background:**

Platinum and anti-PD-1 antibodies are the front-line systemic therapy for recurrent or metastatic head and neck squamous cell carcinoma (RM-HNSCC). However, limited data are available on clinical outcomes and appropriate regimens for patients with RM-HNSCC following treatment failure with these agents.

**Patients and methods:**

We retrospectively analyzed the clinical data of patients with RM-HNSCC from 10 Japanese institutions in whom platinum and nivolumab treatment failed.

**Results:**

Of the 480 patients included in the study, 236 were treated with the best supportive care and had a median overall survival of 3.1 months. The remaining 244 patients received salvage-line chemotherapy, which was paclitaxel + cetuximab in 72 (30%), paclitaxel or docetaxel in 89 (36%), and tegafur/gimeracil/oteracil in 48 (20%); the respective objective response rates were 54.9%, 27.9%, and 25.5%, with median progression-free survival of 5.4 months and median overall survival of 13.0 months. Multivariable analysis identified disease stabilization or response on prior nivolumab and paclitaxel + cetuximab as salvage-line chemotherapy to be associated with encouraging progression-free and overall survival.

**Conclusion:**

This study sheds light on clinical outcomes and prognostic factors in patients with RM-HNSCC after failure of platinum and anti-PD-1 antibody therapy. The findings provide essential baseline data for future therapeutic development in salvage-line settings.

## Graphical Abstract

**Graphical Abstract:**
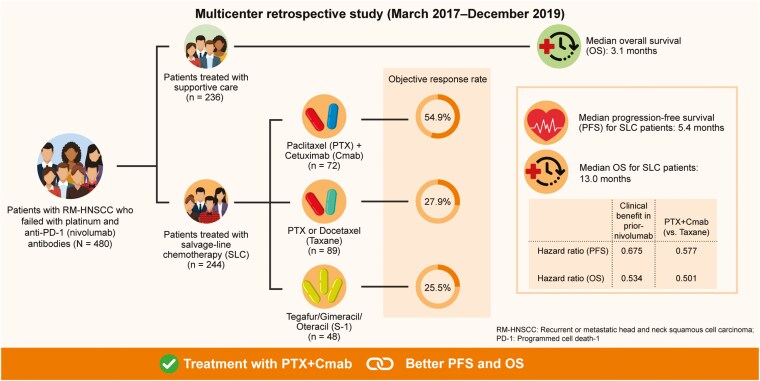

**Implications for practice**

Salvage-line chemotherapy after platinum and anti-PD-1 antibody treatment improved the clinical outcomes of patients with recurrent or metastatic head and neck squamous cell carcinoma. This study is the first report to provide survival data for patients treated with the best supportive care alone and prognostic factors for patients with unified conditions in a salvage-line setting. Disease stabilization or response on prior Nivolumab and treatment with paclitaxel + cetuximab regimen are favorable prognostic factors for PFS and OS. These findings provide essential baseline data for future treatment development in a salvage-line setting for recurrent or metastatic head and neck squamous cell carcinoma.

## Introduction

The advent of cetuximab (Cmab) and immune checkpoint inhibitors (ICIs) has significantly improved clinical outcomes in patients with recurrent or metastatic head and neck squamous cell carcinoma (RM-HNSCC).^1-6^ Based on the results of the CheckMate-141,^7^ KEYNOTE-040^8^ and KEYNOTE-048^9^ trials, platinum and anti-programmed cell death protein 1 (PD-1) antibodies have become key drugs in front-line therapy for RM-HNSCC. As a result of these studies, nivolumab has become the standard treatment for platinum-refractory patients, while pembrolizumab is preferred for platinum-sensitive patients. Chemotherapies induce immune-stimulating changes, including upregulation of major histocompatibility complex molecules and increased presentation of tumor antigens.^10,11^ Long-term data from the KEYNOTE-048 trial^12^ and several other studies^13-17^ reveal that the time to second objective disease progression (PFS2) can be extended by further chemotherapy following treatment with an ICI, with particular emphasis on the utility of taxanes. The extension of PFS2 in the KEYNOTE-048 trial indicated that the survival impact of ICIs was attributed not only to their antitumor effect but also to the enhancement of the effectiveness of subsequent salvage-line chemotherapy (SLC).

However, further advancements in improving the clinical outcomes for patients with RM-HNSCC necessitate the development of new treatment modalities, such as molecular-targeted therapies, next-generation immunotherapies, and antibody-drug conjugates, particularly within the SLC setting. Establishing reliable historical data and identifying prognostic factors in patients who receive the SLC are also essential when conducting clinical trials of new treatment modalities. Despite these advances, limited data are available on clinical outcomes and appropriate treatment regimens for patients with RM-HNSCC in whom front-line platinum and anti-PD-1 antibody therapy have failed. Therefore, we conducted this multicenter retrospective study using clinical data from 10 Japanese institutions to clarify the outcomes and prognostic factors in patients with RM-HNSCC who experienced treatment failure of platinum and nivolumab.

## Materials and methods

### Study design and participants

We retrospectively analyzed the clinical data of patients with RM-HNSCC who had treatment failure of platinum and nivolumab between March 2017 and December 2019 across 10 Japanese institutions (National Cancer Center Hospital, Hokkaido University Hospital, Miyagi Cancer Center, Cancer Institute Hospital of Japanese Foundation for Cancer Research, Yokohama City University Hospital, Tokai University Hospital, Shizuoka Cancer Center, Aichi Cancer Center Hospital, Kobe University Hospital, and Nagasaki University Hospital). In this study, failure of platinum was defined as tumor progression or recurrence within 6 months after the last dose of platinum-containing chemotherapy. Therefore, the patients who experienced recurrence within 6 months after completion of definitive or adjuvant platinum-based chemoradiotherapy were also included as the subjects of this study. In Japan, nivolumab was approved for the treatment of RM-HNSCC in March 2017, specifically for use after platinum failure. This policy ensured that all patients had received platinum before nivolumab.

We collected clinical information on age, follow-up period for censored cases, sex, smoking history, performance status (PS), primary site, viral infection status, metastatic sites, presence or absence of local recurrence, reasons for discontinuation of platinum (refractoriness or intolerance), presence or absence of prior use of Cmab, efficacy of prior nivolumab, the SLC regimen after nivolumab, efficacy of SLC, and survival outcomes. We also collected clinical data for patients treated with best supportive care (BSC) after failure of treatment with nivolumab. Disease stabilization or response on prior nivolumab was defined as disease control, encompassing complete response, partial response (response), or stable disease (stabilization) as the maximum response. Non-CR/Non-PD was defined as patients experiencing clinically meaningful efficacy from nivolumab without disease progression for more than 8 weeks. The study protocol was approved by the Institutional Review Board at each participating institution, and patients and their families were provided with the opportunity to opt-out.

### Statistical analysis

PS was evaluated according to the Eastern Cooperative Oncology Group (ECOG) criteria. Overall survival (OS) was defined as the interval from the date of nivolumab failure to death from any cause or the end of observation. Progression-free survival (PFS) was defined as the interval from the date of starting the SLC to disease progression, death, or end of observation if disease progression did not occur. The objective response rate (ORR) and disease control rate (DCR) were evaluated according to the Response Evaluation Criteria in Solid Tumors 1.1.^18^ Survival functions were estimated using the Kaplan-Meier method and compared using the log-rank test. Univariable and multivariable analyses for the detection of prognostic factors were performed using Cox regression models. All statistical analyses were conducted using SAS software (version 9.4 SAS Institute, Inc.).

## Results

### Patient characteristics

**Figure 1.**
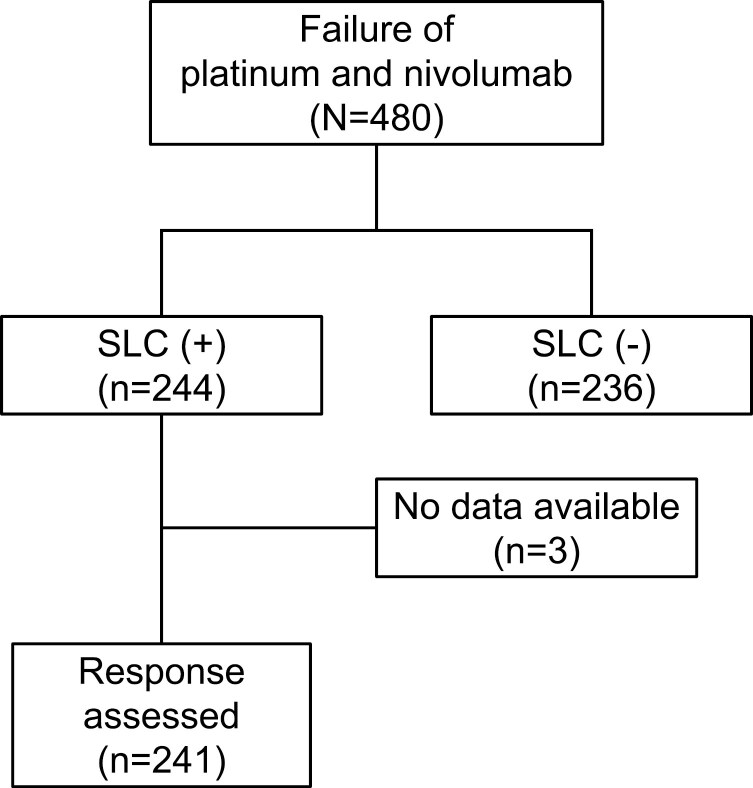
CONSORT diagram. Abbreviation: SLC, salvage-line chemotherapy.

A total of 480 patients were included in the study, of whom 236 received BSC [SLC (−) group] and 244 received the SLC [SLC (+) group]. After excluding 3 patients without tumor response data, the response to the SLC was assessed in 241 patients in the SLC (+) group (Figure 1).

The patient characteristics are shown in Table 1. HPV testing was conducted in 188 cases, of which 44 were HPV-positive. All 44 HPV-positive cases had oropharyngeal primary tumors, including 23 cases in the SLC (+) group and 21 cases in the SLC (−) group.

**Table 1.** Characteristics of all patients and the SLC (-) and SLC (+) groups

Variable	All patients, N = 480 (%)	SLC (-) group, n = 236 (%)	SLC (+) group, n = 244 (%)
Age, years, median (range)	65 (21–89)	65 (22–89)	65 (21–80)
≥65/<65	254 (53)/226 (47)	128 (54)/108 (46)	126 (52)/118 (48)
Follow-up period, days, median (range)	627 (0–1349)	592 (0–1226)	655 (11–1349)
Sex			
Male/female	396 (83)/84 (17)	194 (82)/42 (18)	202 (83)/42 (17)
Smoking history			
Yes/no/unknown	369(76)/110(23)/1(1)	179(76)/56(23)/1(1)	190(78)/54(22)
ECOG performance status			
0/1/2/	76 (16)/239 (50)/55 (12)/	8 (3)/86 (36)/32 (14)/	68 (28)/153 (63)/23 (9)/
3/4/Unknown	54 (11)/6 (1)/50 (10)	54 (23)/6 (3)/50 (21)	0/0/0
Primary site			
Oral cavity	107 (22)	57 (24)	50 (21)
Nasal cavity and paranasal sinus	33 (7)	15 (6)	18 (7)
Nasopharynx	32 (7)	7 (3)	25 (10)
Oropharynx	87 (18)	47 (20)	40 (16)
Hypopharynx	149 (31)	70 (30)	79 (32)
Larynx	51 (11)	27 (11)	24 (10)
Ear canal	10 (2)	5 (2)	5 (2)
Salivary gland	5 (1)	4 (2)	1 (1)
Other	6 (1)	4 (2)	2 (1)
Viral infection status			
EBV/HPV/negative	14 (3)/44 (9)/422 (88)	2 (1)/23 (10)/211 (89)	12 (5)/21 (9)/211 (86)
Metastatic sites, n			
1/≥2/Unknown	152 (32)/278 (58)/50 (10)	57 (24)/129 (55)/50 (21)	95 (39)/149 (61)/0
Local recurrence			
Yes/no/unknown	252 (53)/178 (37)/50 (10)	107 (45)/79 (34)/50 (21)	145 (60)/99 (40)/0
Reasons for discontinuation of platinum-based chemotherapy			
Refractory/intolerant	397 (83)/83 (17)	189 (80)/47 (20)	208 (85)/36 (15)
Prior use of Cmab			
Yes/no	278 (58)/202 (42)	149 (63)/87 (37)	129 (53)/115 (47)
Reason for discontinuation of nivolumab			
Refractory/intolerant/unknown	375 (78)/102 (21)/3 (1)	187 (79)/49 (21)/0	188 (77)/53 (22)/3 (1)
Best response to prior nivolumab			
CR	21 (4)	14 (6)	7 (3)
PR	76 (16)	23 (10)	53 (22)
SD	110 (23)	50 (21)	60 (25)
Non-CR/non-PD	9 (2)	4 (2)	5 (2)
PD	263 (55)	145 (61)	118 (48)
Not evaluated	1 (0)	0	1 (0)
Incidence of irAE due to nivolumab			
Yes/no	126 (26)/354 (74)	57 (24)/179 (76)	69 (28)/175 (72)
SLC regimen			
PTX + Cmab	72 (30)	-	72 (30)
Taxane	89 (36)	-	89 (36)
S-1	48 (20)	-	48 (20)
Other	35 (14)	-	35 (14)

Cmab, cetuximab; CR, complete response; CTx, chemotherapy; EBV, Epstein–Barr virus; ECOG, Eastern Cooperative Oncology Group; HPV, human papillomavirus; irAE, immune-related adverse events; PD, progressive disease; PR, partial response; PTX, paclitaxel; S-1, tegafur/gimeracil/oteracil; SD, stable disease; SLC, salvage-line chemotherapy; Taxane, paclitaxel or docetaxel.

**Figure 2.**
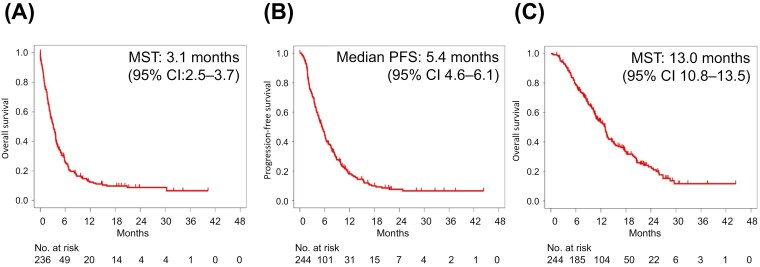
Survival outcomes. (A) Overall survival in patients who did not receive the salvage-line chemotherapy. (B) PFS in patients who received the salvage-line chemotherapy. (C) Overall survival in patients who received the salvage-line chemotherapy. Abbreviations: CI, confidence interval; MST, median survival time; PFS, progression-free survival.

Notably, in the SLC (−) group, 60 patients (25%) had an ECOG PS of 3–4 and the median OS was 3.1 months (95% confidence interval [CI]: 2.5–3.7 months) (Figure 2a). In the SLC (−) group, palliative radiotherapy aimed at symptom relief was administered to 3 out of 236 patients (1%).

In the SLC (+) group, 221 patients (91%) had an ECOG PS of 0-1 and 125 (52%) had achieved stable disease on prior nivolumab. The SLC regimens included paclitaxel + cetuximab [PTX + Cmab] in 72 patients (30%), paclitaxel (PTX) or docetaxel (DTX) [Taxane] in 89 (36%), tegafur/gimeracil/oteracil [S-1] in 48 (20%), and other agents in 35 (14%). Other agents included gemcitabine, S-1 + Cmab, cisplatin, and carboplatin. Of the 244 patients who received the SLC, 97 underwent subsequent chemotherapy following the SLC failure.

### Effectiveness of salvage-line chemotherapy

In the SLC (+) group, the ORR was 36.6% (87/241) with a DCR of 66.4% (160/241). The ORR and DCR were 54.9% and 81.7%, respectively, for PTX + Cmab, 27.9% and 60.9% for Taxane, and 25.5% and 52.1% for S-1 (Table 2). Median PFS was 5.4 months (95% CI 4.6–6.1) and median OS was 13.0 months (95% CI 10.8–13.5) in this group (Figure 2b and c). Median PFS was 6.4 months and OS was 15.4 months for PTX + Cmab, 4.9 months and 10.8 months, respectively, for Taxane, and 4.1 months and 10.4 months for S-1 (Tables 3 and 4).

**Table 2.** ORR and DCR in patients who received SLC following immune checkpoint inhibitor therapy.

Treatment regimen	ORR	DCR
All SLC (*N* = 241)	36.6% (87/241)	66.4% (160/241)
PTX + Cmab (*n* = 71)	54.9% (39/71)	81.7% (58/71)
Taxane (*n* = 87)	27.9% (24/87)	60.9% (53/87)
S-1 (*n* = 48)	25.5% (12/48)	52.1% (25/48)
Other (*n* = 35)	35.3% (12/35)	68.6% (24/35)

Abbreviations: DCR, disease control rate; ORR, objective response rate; PTX + Cmab, paclitaxel plus cetuximab; S-1, tegafur/gimeracil/oteracil; SLC, salvage-line chemotherapy; Taxane, paclitaxel or docetaxel.

**Table 3.** Prognostic analysis of median PFS in patients who received chemotherapy following immune checkpoint inhibitor therapy

Covariate	Median PFS			Univariable analysis (n = 244)				Multivariable analysis (n = 244)			
	Estimate	95% CI		HR	95% CI		*P*-value	HR	95% CI		*P*-value
Age, years											
<65	5.4	3.9	6.8	Reference				Reference			
≥65	5.2	4.3	6.1	1.03	0.782	1.361	0.82	0.95	0.706	1.280	0.74
ECOG PS											
0	5.4	3.9	8.2	Reference				Reference			
1–2	5.5	4.3	6.1	1.15	0.839	1.572	0.39	1.13	0.811	1.575	0.47
Primary site											
Oropharynx/larynx	5.2	3.8	6.8	Reference				Reference			
Oral cavity/hypopharynx	5.2	4.3	6.0	0.95	0.680	1.318	0.74	1.06	0.712	1.571	0.78
Nasopharynx	9.9	4.8	13.0	0.64	0.383	1.077	0.093	0.96	0.464	1.987	0.91
Other	4.0	2.5	8.4	1.09	0.669	1.783	0.72	1.25	0.732	2.139	0.41
Viral infection status											
Negative	5.2	4.3	5.9	Reference				Reference			
EBV	10.7	3.0	15.2	0.53	0.273	1.047	0.068	0.59	0.237	1.446	0.25
HPV	5.3	2.1	8.0	1.27	0.778	2.072	0.34	1.30	0.730	2.321	0.37
Metastatic sites, n											
1	5.7	3.9	7.7	Reference				Reference			
≥2	5.2	4.3	6.1	1.03	0.773	1.366	0.85	0.95	0.706	1.266	0.71
Local recurrence											
No	5.7	4.5	6.3	Reference				Reference			
Yes	5.2	3.9	6.2	1.10	0.828	1.456	0.52	1.14	0.852	1.536	0.37
Disease stabilization or response on prior nivolumab											
No	4.9	3.5	6.0	Reference				Reference			
Yes	5.9	4.6	7.6	0.67	0.507	0.887	0.005	0.68	0.502	0.907	**0.009**
SLC regimen											
Taxane	4.9	3.6	5.5	Reference				Reference			
PTX + Cmab	6.4	4.7	8.5	0.63	0.443	0.894	0.01	0.58	0.402	0.828	**0.003**
S-1	4.1	2.2	8.3	0.98	0.675	1.427	0.92	0.90	0.615	1.328	0.60
Other	6.3	3.4	8.0	0.77	0.499	1.174	0.22	0.89	0.552	1.444	0.64

Bold font indicates statistical significance at *P*<0.01. CI, confidence interval; EBV, Epstein–Barr virus; ECOG, Eastern Cooperative Oncology Group; HPV, human papillomavirus; HR, hazard ratio; PFS, progression-free survival; PS, performance status; PTX + Cmab, paclitaxel plus cetuximab; S-1, tegafur/gimeracil/oteracil; SLC, salvage-line chemotherapy; Taxane, paclitaxel or docetaxel.

**Table 4.** Prognostic analysis of median OS in patients who received chemotherapy following immune checkpoint inhibitor therapy

Covariate	Median OS			Univariable analysis (n = 244)				Multivariable analysis (n = 244)			
	Estimate	95% CI		HR	95% CI		*P*-value	HR	95% CI		*P*-value
Age, years											
<65	11.9	10.3	13.4	Reference				Reference			
≥65	13.4	10.8	16.2	0.86	0.631	1.165	0.33	0.77	0.554	1.070	0.12
ECOG PS											
0	13.5	12.3	20.5	Reference				Reference			
1–2	11.9	9.9	13.4	1.48	1.029	2.128	0.034	1.45	0.990	2.119	**0.056**
Primary site											
Oropharynx/larynx	15.0	13.1	20.5	Reference				Reference			
Oral cavity/hypopharynx	10.7	8.5	12.8	1.53	1.054	2.208	0.025	1.51	0.963	2.361	**0.073**
Nasopharynx	15.2	10.4	.	0.77	0.420	1.418	0.41	0.94	0.399	2.224	0.89
Other	16.8	6.3	23.4	1.10	0.623	1.932	0.75	1.09	0.579	2.048	0.79
Viral infection status											
Negative	12.5	10.4	13.4	Reference				Reference			
EBV	18.0	9.5	.	0.52	0.243	1.120	0.095	0.80	0.265	2.430	0.70
HPV	20.1	8.9	25.3	0.75	0.437	1.275	0.29	0.97	0.512	1.855	0.94
Metastatic sites, n											
1	13.8	12.8	18.0	Reference				Reference			
≥2	10.8	10.2	13.3	1.32	0.957	1.821	0.091	1.13	0.808	1.571	0.48
Local recurrence											
No	13.0	10.4	15.4	Reference				Reference			
Yes	13.0	10.4	13.8	1.04	0.762	1.416	0.81	1.16	0.834	1.600	0.39
Disease stabilization or response on prior nivolumab											
No	10.3	8.7	11.3	Reference				Reference			
Yes	15.2	13.1	20.5	0.52	0.382	0.712	<.001	0.53	0.385	0.740	**<.001**
SLC regimen											
Taxane	10.8	9.5	13.1	Reference				Reference			
PTX + Cmab	15.4	13.0	26.0	0.57	0.381	0.848	0.006	0.50	0.332	0.757	**0.001**
S-1	10.4	7.5	13.5	1.07	0.713	1.595	0.755	0.95	0.629	1.441	0.82
Other	13.4	10.4	20.6	0.63	0.391	1.016	0.058	0.73	0.431	1.243	0.25

Bold underline font indicates statistical significance at *P* < 0.01. CI, confidence interval; EBV, Epstein–Barr virus; ECOG, Eastern Cooperative Oncology Group; HPV, human papillomavirus; HR, hazard ratio; OS, overall survival; PS, performance status; PTX + Cmab, paclitaxel plus cetuximab; SLC, salvage-line chemotherapy; S-1, tegafur/gimeracil/oteracil; Taxane, paclitaxel or docetaxel.

### Prognostic factors in patients receiving salvage-line chemotherapy

Univariable and multivariable analyses included age, ECOG PS, primary site, viral infection status, number of metastatic sites, presence or absence of local recurrence, disease stabilization or response on prior nivolumab, and the SLC regimen as covariates.

Multivariable analysis revealed that disease stabilization or response on prior nivolumab (PFS, hazard ratio [HR] 0.68, 95% CI, 0.502-0.907, *P* = .009; OS, HR 0.53, 95% CI, 0.385-0.740, *P* < .001) and treatment with PTX + Cmab (PFS, HR 0.58, 95% CI, 0.402-0.828, *P* = .003 [vs Taxane]; OS, HR 0.50, 95% CI, 0.332-0.757, *P* = .001 [vs Taxane]) were associated with better PFS and OS. An ECOG PS of 1-2 (HR 1.45, 95% CI, 0.990-2.119, *P* = .056) and primary site in the oral cavity/hypopharynx (HR 1.51, 95% CI, 0.963-2.361, *P* = 0.073 [vs oropharynx/larynx]) showed a marginal association with worse OS (Tables 3 and 4).

## Discussion

This large-scale study provides real-world data on clinical outcomes in patients with RM-HNSCC after failure of platinum and nivolumab. Additionally, this study offers survival data for patients treated with BSC in a salvage-line setting (median OS 3.1 months), which is rarely reported. However, this data may be subject to a potential selection bias, as most patients treated with BSC might be in miserable situations without the indication of the SLC.

Our data also included a distinct population of patients who achieved long-term survival without the SLC. These patients were likely nivolumab responders who discontinued nivolumab due to toxicity or clinical judgment rather than disease progression. In contrast, the median OS was 13.0 months in patients who received the SLC, consistent with survival data from the EXTREME study, which was conducted in the first-line setting.^19^ This suggests that ICIs have dramatically improved the prognosis of RM-HNSCC. There is a subset of patients who have shown long-term efficacy from the treatment, contributing to their extended survival.

Our study also demonstrated the clinically meaningful efficacy of the SLC, with an ORR of 36.6% and a median PFS of 5.4 months. In the CheckMate-141,^7^ KEYNOTE-040,^8^ and EAGLE^20^ trials, the standard arms included monotherapy with methotrexate, DTX, Cmab, and fluoropyrimidine. The ORR, median PFS, and median OS in the standard arms ranged 5.8%-17.3%, 2.0-3.7 months, and 5.1-8.3 months, respectively.^7,8,20^ Against ICIs, the HR for OS was 1.33-1.56 for methotrexate, 1.16-1.22 for DTX, and 1.79-2.13 for Cmab.^7,8^ The ORR, median PFS, and median OS in the second-line setting were 43.3%, 6.2 months, and 8.5 months, respectively, for PTX^21^; 24.2%, 4.8 months, and 7.3 months for capecitabine^22^; and 6%, 1.7 months, and 6.0 months for methotrexate.^23^

Several recent studies have suggested that prior exposure to ICIs may enhance the efficacy of subsequent chemotherapy, a phenomenon observed in other cancers such as non-small cell lung cancer^24^ and advanced esophageal squamous cell carcinoma.^25^ In our study, the enhanced chemotherapy efficacy may also be due to the immunomodulatory effects of ICIs. The ORR was 53.4% in patients with non-small cell lung cancer and 37.5% in patients with advanced esophageal squamous cell carcinoma. The immunomodulatory effects of ICIs in the tumor microenvironment could contribute to this enhanced sensitivity to chemotherapy observed in these patients.^26-28^ The potential mechanisms underlying this enhanced response to chemotherapy include the direct cytotoxic effects of taxanes on tumor cells and their ability to modulate the immune response. Taxanes have been shown to reduce the number of regulatory T-cells and activate toll-like receptors on immune cells, promoting an anti-tumor immune response. This dual action of taxanes could explain the improved outcomes observed in our study.^29,30^

Although evidence of the effectiveness of Cmab monotherapy remains limited, the INTERLINK-1 study,^31^ which was a randomized controlled trial comparing the efficacy of monalizumab + Cmab versus Cmab monotherapy in patients with RM-HNSCC previously treated with platinum and an anti-PD-1 antibody, reported an ORR of 23.9%, median PFS of 3.8 months, and median OS of 8.6 months in patients on Cmab monotherapy. Therefore, taxane monotherapy, whether with PTX or DTX, is relatively effective in comparison with other agents.

In terms of the appropriate treatment regimen, we found that patients who received PTX + Cmab had an ORR of 54.2%, median PFS of 6.4 months, and median OS of 15.4 months, suggesting encouraging clinical outcomes. Saleh et al investigated the effectiveness of chemotherapy after ICI therapy and reported that patients who received the SLC after failure on an ICI had an ORR of 30% and a median PFS of 3.6 months.^13^ In their study, the ORR was higher in patients treated with a taxane + Cmab than in those treated with other regimens (53% vs 25%, *P* = .024). A multicenter prospective phase II trial that evaluated the efficacy and safety of paclitaxel + biweekly Cmab in patients with RM-HNSCC previously treated with both platinum and an anti-PD-1 antibody reported an ORR of 68.7% (95% CI 49.9-83.8), a median PFS of 5.7 months, and a median OS of 13.4 months.^32^ Our results regarding the efficacy of treatment with PTX + Cmab are consistent with those previously reported.

Our multivariable analysis also identified ECOG PS of 1-2 and the primary site in the oral cavity/hypopharynx to be marginal predictors of worse OS. Prognostic analysis of the results of 2 phase III trials in RM-HNSCC (E1393 and E1395) revealed that ECOG PS of 1 (HR 1.45, 95% CI, 1.15-1.83, *P* = 0.0016 [vs 0]) and primary tumor located in the oral cavity/hypopharynx (HR 1.32, 95% CI, 1.06-1.64, *P* = 0.011 [vs others]) were associated with worse OS,^33^ which is consistent with our findings. Moreover, our study revealed that disease stabilization or response on prior nivolumab was a favorable prognostic factor for PFS and OS. A previous study found that over 50% of PD-1 receptors on T-cells remained occupied even 32 weeks after discontinuation of nivolumab. This enduring occupancy rate may explain the persistent immunomodulatory effect of nivolumab and suggests that this agent has a prolonged influence on T-cell function beyond what its pharmacokinetic profile would suggest.^34^ Saleh et al found that disease stabilization or response on prior ICI therapy was a predictor of better PFS (HR 0.56, 95% CI, 0.315-0.980) and OS (HR 0.51, 95% CI, 0.268-0.970),^13^ which is also consistent with our results.

This study has some limitations, primarily due to its retrospective design, inconsistent disease monitoring intervals, and varying criteria used to assess treatment effects across different facilities. These drawbacks may have affected the accuracy of our data regarding disease stabilization or response on prior nivolumab and ORR and PFS for the SLC. Additionally, patients who received nivolumab as a first-line setting (recurrence within 6 months after definitive or adjuvant chemoradiotherapy) were more likely to receive PTX + Cmab as the SLC, while patients who administered nivolumab as a later-line setting tended to be treated with the SLC not included Cmab because most of them were previously treated with Cmab-based regimen. Furthermore, patients who received nivolumab as a first-line setting had another treatment option of fluoropyrimidine after failure to PTX + Cmab, which might influence the better treatment outcomes of PTX + Cmab arm.

Nevertheless, OS remains a robust endpoint and a pivotal metric in clinical trials, especially in the SLC setting. Despite these potential sources of bias, we believe that this study provides valuable historical data that can serve as a reliable benchmark for future clinical trials in patients with RM-HNSCC.

## Conclusion

This large-scale study has provided valuable real-world data on clinical outcomes and prognostic factors in patients with RM-HNSCC who have failed on platinum and nivolumab. Its findings serve as a pivotal historical reference for future developments in therapy, especially in the salvage-line setting.

## Data Availability

The datasets used and/or analyzed during this study are available from the corresponding author upon reasonable request.
